# Supplementary material for: Epidemiology of Shiga Toxin-Producing Escherichia coli O157 in the Province of Alberta, Canada, 2009–2016
Source: Toxins (Basel). 2019 Oct 22;11(10):613. doi: 10.3390/toxins11100613 (PMC6832344; doi:10.3390/toxins11100613)
Supplement: Supplementary file 1 [file toxins-11-00613-s001.pdf]

Supplementary Materials: Epidemiology of Shiga Toxin-Producing *Escherichia coli* O157 in the Province of Alberta, Canada, 2009–2016

Luiz F. Lisboa, Jonas Szelewicki, Alex Lin, Sarah Latonas, Vincent Li, Shuai Zhi, Brendon D. Parsons, Byron Berenger, Sumana Fathima and Linda Chui

Table S1. PCR primers and probes utilized in the study.

	Sequence (5'–3')	Reference
*stx*1-F	CATCGCGAGTTGCCAGAAT	
*stx*1-R	GCGTAATCCCACGGACTCTTC	Chui [38]
*stx*1-P	FAM-CTGCCGGACACATAGAAGGAAACTCATCA-TAMRA	
*stx*2-F	CCGGAATGCAAATCAGTC	
*stx*2-R	CAGTGACAAAACGCAGAACT	Chui [38]
*stx*2-P	FAM-ACTGAACTCCATTAACGCCAGATATGA-TAMRA	
*stx*1a-F	CCTTTCCAGGTACAACAGCGGTT	Scheutz [6]
*stx*1a-R2	GGAAACTCATCAGATGCCATTCTGG	
*stx*1a-F	CTACGGCTTATTGTTGAACGAAAT	
*stx*1a-R	GCTGTAACGTGGTATAGCTACTG	Zhi [40]
*stx*1a-P	TT+TC+C+A+GG+T+AC	
*stx*2a-F	GCGATACTG**R**G**B**ACTGTGGCC	
*stx*2a-R	CCG**K**CAACCTTCACTGTAAATGTG	Scheutz [6]
*stx*2a-P	GCCACCTTCACTGTGAATGTG	
*stx*2a-F	CTGTTAATGCAATGGCGGC	
*stx*2a-R	GCAAATCCGGAGCCTGA	Zhi [40]
*stx*2a-P	AG+G+AT+G+A+C+ACAT	
*stx*2c-F	GAAAGTCACAGTTTTTATATACAACGGGTA	Scheutz [6]
*stx*2c-R	CCGGCCAC**Y**TTTACTGTGAATGTA	
eae-F	CATTGATCAGGATTTTTCTGGTGATA	
eae-R	CTCATGCGGAAATAGCCGTTA	Nielsen [37]
eae-P	FAM-ATAGTCTCGCCAGTATTCGCCACCAATAC-BHQ	

'+' denotes a locked nucleic acid (LNA). Bold-face letters denote ambiguous nucleotides.
